# Risk Factors for Colonization with Extended-Spectrum β-Lactamase–producing Bacteria and Intensive Care Unit Admission

**DOI:** 10.3201/eid1308.070071

**Published:** 2007-08

**Authors:** Anthony D. Harris, Jessina C. McGregor, Judith A. Johnson, Sandra M. Strauss, Anita C. Moore, Harold C. Standiford, Joan N. Hebden, J. Glenn Morris

**Affiliations:** * University of Maryland, Baltimore, Maryland, USA; † Veterans Affairs Maryland Health Care System, Baltimore, Maryland, USA; ‡ University of Maryland Medical Center, Baltimore, Maryland, USA

**Keywords:** Drug-resistance microbial, risk factors, intensive care units, beta lactamases, *Escherichia coli*, *Klebsiella*, research

## Abstract

Extended-spectrum β-lactamase (ESBL)–producing bacteria are emerging pathogens. To analyze risk factors for colonization with ESBL-producing bacteria at intensive care unit (ICU) admission, we conducted a prospective study of a 3.5-year cohort of patients admitted to medical and surgical ICUs at the University of Maryland Medical Center. Over the study period, admission cultures were obtained from 5,209 patients. Of these, 117 were colonized with ESBL-producing *Escherichia coli* and *Klebsiella* spp., and 29 (25%) had a subsequent ESBL-positive clinical culture. Multivariable analysis showed the following to be statistically associated with ESBL colonization at admission: piperacillin-tazobactam (odds ratio [OR] 2.05, 95% confidence interval [CI] 1.36–3.10), vancomycin (OR 2.11, 95% CI 1.34–3.31), age >60 years (OR 1.79, 95% CI 1.24–2.60), and chronic disease score (OR 1.15; 95% CI 1.04–1.27). Coexisting conditions and previous antimicrobial drug exposure are thus predictive of colonization, and a large percentage of these patients have subsequent positive clinical cultures for ESBL-producing bacteria.

## Abstract 2

Coexisting conditions and previous antimicrobial drug exposure predict colonization.

Extended-spectrum β-lactamase (ESBL)–producing gram-negative bacteria are emerging pathogens. Clinicians, microbiologists, infection control practitioners, and hospital epidemiologists are concerned about ESBL-producing bacteria because of the increasing incidence of such infections, the limitations of effective antimicrobial drug therapy, and adverse patient outcomes (*1**–**5*).

Research conducted to date has focused on identifying risk factors for colonization with multidrug-resistant, gram-positive bacteria. In contrast, little research has been conducted to identify the risk factors for colonization with gram-negative multidrug-resistant bacteria in nonoutbreak settings. To our knowledge, no study of the magnitude of our study has been conducted, nor have any studies based in the United States sought to identify risk factors for colonization with ESBL-producing bacteria on admission to an intensive care unit (ICU).

The primary objective of our study was to identify factors predictive of colonization with ESBL-producing bacteria at admission to an intensive care unit (ICU). In addition, we identified the percentage of patients colonized with ESBL-producing bacteria who had a subsequent positive clinical culture for the same species of ESBL-producing bacteria. Understanding risk factors for colonization is important for several reasons. First, understanding the potential causal mechanisms of colonization can lead to successful infection control, involving antimicrobial stewardship and public health interventions aimed at controlling the emergence of ESBL-producing bacteria. Second, such knowledge can help identify which patients should be receiving empiric ESBL-targeted antimicrobial therapy. Some hospitals have used active surveillance culturing for antimicrobial drug–resistant, gram-negative bacteria to help guide empiric therapy (*6*).

## Materials and Methods

### Study Population and Sample Collection

We conducted a prospective cohort study of patients admitted to either the surgical or medical ICU at the University of Maryland Medical Center from September 1, 2001, through June 1, 2005. Descriptions of the hospital and the ICUs are reported in other publications (*7**,**8*). During the study period, on average, 8.6 clinical cultures per month were positive for ESBL-producing bacteria. No outbreaks of ESBL-producing bacteria were found among clinical cultures based on control process charting. No additional infection control precautions were used for patients with ESBL-producing bacteria on clinical culture. ESBL surveillance culture results were not given to physicians or nurses. Contact isolation precautions were applied for patients with vancomycin-resistant enterococci or methicillin-resistant *Staphylococcus aureus* infections.

During the study period, nurses obtained perianal specimens for culture from all ICU patients within 48 hours of ICU admission. All patients who had admission culture results were included in this study. Patients with multiple admissions to either of the ICUs during the study period were allowed to enter the cohort of at-risk patients multiple times, as long as they were not positive for ESBL-producing bacteria on any prior admissions (because patients remain at risk for ESBL-producing bacteria on each subsequent admission). This study was approved by the Institutional Review Board of the University of Maryland, Baltimore. Informed consent was not required by the Institutional Review Board because perianal specimens were cultured as part of infection control quality improvement involving active surveillance culturing for vancomycin-resistant enterococci.

### Microbiologic Methods

The perianal cultures were processed for ESBL-producing bacteria in real time as the specimens were collected. The perianal cultures were first screened for potential ESBL-producing bacteria by plating onto MacConkey agar (Remel, Lenexa, KS, USA) with 2 μg/mL of ceftazidime added to the cooled agar before the plates were poured (*9*). Plates were incubated at 37°C for 24 to 48 hours. Lactose-fermenting colonies growing on the ceftazidime-containing plates were identified as *Escherichia coli* or *Klebsiella* species by using API 20E identification strips (bioMérieux Vitek, Inc., Hazelwood, MO, USA). All *E. coli* and *Klebsiella* isolates underwent ESBL confirmatory testing by disk diffusion for ceftazidime and cefotaxime with and without clavulanic acid as recommended by the Clinical Laboratory Standards Institute’s guidelines (*10*).

### Data Collection and Variables

For all patients included in the study, we collected data regarding the patient’s previous hospital antimicrobial drug exposures, length of hospitalization before ICU admission, coexisting conditions, previous positive cultures, and other hospitalization-related and demographic information. Antimicrobial drug exposures were assessed in the period between hospital admission and ICU admission. Antimicrobial drugs were analyzed as binary variables; if an antimicrobial drug was received during the period defined above, it was classified as having been received independent of the number of doses received. Duration of antimicrobial drug exposure was not analyzed. Coexisting conditions were assessed by the Charlson Comorbidity Index, the Chronic Disease Score (CDS), and the infectious disease–specific CDS (CDS-ID) (*11**–**13*).

Initial bivariable statistical comparisons were conducted by using the χ^2^ test for categorical data and the Student *t* test or Wilcoxon test for continuous data. Continuous variables that were not normally distributed were categorized for the purpose of multivariable analyses. To identify patient characteristics associated with colonization by an ESBL-producing bacterium on ICU admission, we used multivariable logistic regression. Because patients were allowed to enter into the study multiple times, we also assessed the need to control for the correlated error structure of the data. All variables that were associated with ESBL colonization in the bivariable analysis at the p<0.1 level were included in the model-building stages of the multivariable analysis. A stepwise model building method was used. Variables were retained in the final model if they were significant at a p<0.05 level or if they were observed to have a confounding effect on the association between another predictor and ESBL colonization status. A confounding effect was defined as a change in the model coefficient by >10%. An additional bivariable statistical analysis was performed to identify risk factors for subsequent clinical culture positivity with the same species of ESBL-producing bacteria among the cohort of patients colonized with an ESBL-producing bacteria. We calculated the C statistic of the final model. The C statistic reports values from 0.5 (indicating no predictive power) to 1.0 (indicating perfect prediction). In addition, we calculated the sensitivity, specificity, positive predictive value, and negative predictive value for patients with or without all dichotomous variables in the final model. Statistical analysis was performed with SAS Version 9.1 (SAS Institute, Cary, NC, USA).

## Results

During the study period, 5,209 (84%) admitted patients had results of admission perianal cultures and were included in this study, 4,398 patients had 1 ICU admission, and 618 patients had repeat admissions. Ninety-one percent of the surveillance cultures were obtained within the first 12 hours of ICU admission. The cross-sectional patient cohort consisted of 2,096 (40%) admissions to the medical ICU and 3,113 (60%) admissions to the surgical ICU. The mean age of the patients was 55 years. The mean comorbidity score as measured by the CDS–ID was 2.73 and 2.42 as measured by the Charlson Comorbidity Index. Based upon International Classification of Diseases, 9th revision (ICD-9) codes, 1,285 (25%) had diabetes, 1,344 (26%) had cancer, and 193 (4%) were HIV positive; 1,594 (31%) of patients had been transferred from another healthcare facility, and 1,693 (33%) had been previously admitted to the same hospital within the past year.

We examined patient characteristics, coexisting conditions, and previous antimicrobial drug exposures to identify factors potentially associated with colonization by an ESBL-producing bacterium on ICU admission (Table 1, bivariable analysis). Of 5,209 patient admissions, 117 (2%) patients were colonized by an ESBL-producing *E. coli* or *Klebsiella* species bacterium on ICU admission. Specifically, 76 (65%) patients were colonized by an ESBL-producing *E. coli,* 55 (47%) were colonized by an ESBL-producing *Klebsiella* species, and 14 (12%) patients were colonized by both. Stratified bivariable analyses by organism are as follows: for *E. coli*, zosyn (odds ratio [OR] 1.93; 95% confidence interval [CI] 1.17–3.18), vancomycin (OR 1.66, 95% CI, 0.91–3.03), age (OR 2.51, 95% CI 1.57–4.00), and coexisting conditions (OR 1.19, 95% CI 1.05–1.35); for *Klebsiella* spp., zosyn (OR 2.30, 95% CI 1.31–4.06), vancomycin (OR 3.91, 95% CI 2.21–6.91), age (OR 1.29, 95% CI 0.76–2.20), and coexisting conditions, as measured by CDS-ID (OR 1.12, 95% CI 0.96–1.31). Stratified analysis results for those patients who were in the hospital at least 24 hours before ICU admission are as follows: zosyn (OR 2.34, 95% CI, 1.11–4.94), vancomycin (OR 3.25, 95% CI 1.57–6.75), age (OR 1.39, 95% CI 0.68–2.86), and coexisting conditions as measured by CDS-ID (OR 95% CI 1.01–1.44).

**Table 1:** Potential predictors of colonization by an ESBL-producing bacterium on ICU admission*

Potential predictor	No. ESBL colonized (n = 117)	No. not ESBL colonized (n = 5,092)	p value†
Age, y (median, IQR)	62 (49–71)	56 (45–67)	<0.01
CDS (median, IQR)	8 (5–10)	8 (5–10)	0.20
CDS-ID (median, IQR)	3.21 (1.83–4.78)	2.83 (1.83–3.40)	<0.01
Sex, female, no. (%)	59 (50)	2,311 (45)	0.30
Antimicrobial drug exposures, no. (%)‡			
Quinolone	18 (15)	617 (12)	0.32
1st-generation cephalosporin	9 (8)	559 (11)	0.30
3rd-generation cephalosporin	7 (6)	293 (6)	0.84
Vancomycin	34 (29)	616 (12)	<0.01
Aminoglycoside	11 (9)	366 (7)	0.36
Piperacillin-tazobactam	50 (43)	1,090 (21)	<0.01
Cefepime	9 (8)	161 (3)	0.01
Imipenem	11 (9)	224 (4)	0.02

*ESBL, extended-spectrum β-lactamase; ICU, intensive care unit; IQR, interquartile range; CDS, Chronic Disease Score; CDS-ID, infectious disease–specific CDS. †Fisher exact test for dichotomous predictors and Wilcoxon test for continuous predictors. ‡Antimicrobial drug exposures that occurred during the index hospital admission but before ICU admission.

The results of the final multivariable logistic regression analysis are shown in Table 2. Age >60 years (OR 1.79, 95% CI 1.24–2.60), coexisting conditions as measured by the CDS-ID (OR 1.15, 95% CI 1.04–1.27), in-hospital use of piperacillin-tazobactam (OR 2.05, 95% CI 1.36–3.10), and in-hospital use of vancomycin (OR 2.11, 95% CI 1.34–3.31) were all found to be independently associated with colonization by an ESBL-producing bacterium on admission to an ICU. No other antimicrobial drug was found to have a significant (p<0.05) effect in the final multivariable model. Note that we did not adjust for the correlated error structure of the data in the final analysis; because the correlation was low, this adjustment had little effect on our estimates (data not shown). The C statistic of the final model was 0.69. Patients categorized on the basis of the presence of all of the following dichotomous predictors of the final model (zosyn, vancomycin and age >60) yielded a sensitivity of 9.4%, specificity of 97.3%, positive predictive value of 7.3%, and negative predictive value of 97.9%.

**Table 2:** Independent predictors of ESBL-producing bacteria colonization in multivariable logistic regression model*

Predictor	OR	95% CI
Age >60	1.79	1.24, 2.60
CDS-ID	1.15	1.04, 1.27
Vancomycin†	2.11	1.34, 3.31
Piperacillin-tazobactam†	2.05	1.36, 3.10

*ESBL, extended-spectrum β-lactamase; OR, odds ratio; CI, confidence interval; CDS-ID, infectious disease–specific Chronic Disease Score. †Antimicrobial drug exposures were assessed during the period between hospital admission and intensive care unit admission.

For the 117 patients identified as colonized with ESBL-producing bacteria, we assessed their history of culture positivity with ESBL-producing bacteria as well as other antimicrobial drug–resistant bacteria (Table 3). Of the ESBL-colonized patients, 6 (5%) had positive clinical cultures for ESBL-producing bacteria during the same hospital admission but before ICU admission, and 29 (25%) had a subsequent ESBL-positive clinical culture from the time an ICU admission surveillance specimen was obtained for culture to the date of hospital discharge. The only risk factor that predicted subsequent positive ESBL clinical culture was the amount of time in the hospital between positive surveillance culture and hospital discharge (OR 1.03 per additional day, 95% CI 1.01–1.06). These 29 patients had 56 clinical cultures with ESBL-producing bacteria. The sources of the 56 clinical cultures positive for ESBL-producing bacteria were the following: 9 blood cultures, 17 sputum or bronchoscopy specimens, 10 urine cultures, 12 wound cultures, and 8 miscellaneous sources. Of 117 ESBL-colonized patients, 41 (35%) were known to have been previously infected or colonized with either methicillin-resistant *S. aureus* (MRSA) or vancomycin-resistant enterococci (VRE). Among the 5,092 patients not colonized with ESBL-producing bacteria, 33 (0.6%) had a subsequent positive ESBL clinical culture with the same bacterial species between the time of ICU admission surveillance culture to the date of hospital discharge.

**Table 3:** History of culture positivity with antimicrobial drug–resistant bacteria among 117 patients colonized with ESBL-producing bacteria at ICU admission*

Drug-resistant bacteria	No. ESBL colonized (%)
ESBL-positive clinical cultures before ICU admission†	6 (5)
ESBL-positive clinical cultures after colonization	29 (25)
Methicillin-resistant *Staphylococcus aureus*†	25 (21)
Vancomycin-resistant enterococci‡	27 (23)

*ESBL, extended-spectrum β-lactamase; ICU, intensive care unit. †Positive clinical cultures during the same hospital admission but before ICU admission. ‡Clinical or surveillance cultures at any time before ICU admission.

## Discussion

In this study, we identified risk factors for colonization with ESBL-producing *E. coli* and *Klebsiella* spp. at ICU admission. We identified age >60 years, comorbidity as measured by the CDS-ID, previous in-hospital piperacillin-tazobactam use (current admission), and previous present admission in-hospital vancomycin use (current admission) as independent risk factors. We also quantified the ESBL colonization/clinical culture positivity rate among these patients and addressed the question of whether patients colonized with ESBL had a history of colonization with MRSA and VRE.

The risk factors identified are potentially important because they can help determine which patients may need empiric antimicrobial drug therapy targeted to the ESBL-producing bacteria. Carbapenem antimicrobial agents may be preferred as empiric choice for patients at risk for ESBL-producing bacteria (*2*). We are not recommending that all patients with these risk factors receive empiric antimicrobial drug therapy targeted to ESBL-producing bacteria. However, among particular patients with the identified risk factors and levels of severity of infection that require empiric therapy, a choice of empiric therapy that includes coverage of ESBL-producing bacteria may be warranted. Thus, we recommend that for patients in ICUs with similar characteristics to the units in this study, physicians consider using antimicrobial agents targeted against ESBL-producing bacteria. These ESBL-targeted drugs should be considered when the physician chooses to prescribe an antimicrobial drug for situations such as fever of unknown origin, suspected pneumonia, or suspected bacteremia. In addition, these risk factors identified may be of use to hospital antimicrobial drug stewardship programs and pharmacy and therapeutics committees.

We hope that our risk factor study and other risk factor studies in the area of antimicrobial drug resistance will be used in future antimicrobial agent stewardship intervention studies and future infection control intervention studies. Previous risk factor studies have led to antimicrobial agent stewardship intervention studies aimed at controlling ESBL-producing bacteria (*14**,**15*). In the areas of pneumonia and neutropenia patients with fever, risk factors studies have successfully led to intervention studies that have affected national guidelines (*16**–**18*). Well-designed intervention studies, based on risk factor studies of antimicrobial drug resistance, can lead to more appropriate antimicrobial drug use, which will improve patient outcomes and decrease the emergence of antimicrobial drug resistance (*19**,**20*).

The risk factors identified may be causally related to the outcome of ESBL-colonization or may only be statistically associated. Age >60 years and the presence of coexisting conditions have validity and biologic plausibility for a causal association with colonization status (*1**,**9**,**21*). The identification of piperacillin-tazobactam and vancomycin as risk factors is more intriguing. Vancomycin and piperacillin-tazobactam are widely used at our tertiary-care hospital, the University of Maryland Medical Center, and thus may just be markers of ICU patients who require broad-spectrum antimicrobial coverage. However, understanding intestinal ecology and antimicrobial drug resistance is still in nascent stages. Vancomycin and piperacillin-tazobactam may be true causal risk factors for colonization with ESBL-producing bacteria. Piperacillin-tazobactam is believed to be effective against ESBL-producing bacteria only when the inoculum is low (*22*). Thus, with regard to the intestinal flora, piperacillin-tazobactam may not be effective at eradicating ESBL-producing bacteria due to inoculum effects and low intestinal concentration of piperacillin-tazobactam. Additionally, we were surprised by the identification of piperacillin-tazobactam as a risk factor as some hospitals have adopted antimicrobial drug stewardship policies that have limited the prescribing of cephalosporins and increased the use of antimicrobial drugs, including piperacillin-tazobactam, in an effort to control ESBL-producing bacteria (*15**,**23*). Vancomycin may be a risk factor through relative decolonization of the normal flora through vancomycin exposure and then subsequent colonization with ESBL strains through horizontal transmission before ICU admission (*24*,*25*).

We found a ratio of colonization to clinical culture positivity that was the same order of magnitude as for VRE and MRSA (*26*–*29*). In addition, only 35% of patients with ESBL-colonization were previously known to be VRE or MRSA positive. These numbers and the local prevalence rate of ESBL-producing bacteria are important parameters in assessing the cost-effectiveness of active surveillance for ESBL-producing bacteria. Further work, including cost-effectiveness studies, needs to address whether active surveillance is beneficial for ESBL-producing bacteria.

Several studies. performed worldwide, have analyzed risk factors for colonization with multidrug resistant *Enterobacteriaceae*. Many studies have not analyzed the specific antimicrobial drug resistance mechanism and thus are not directly comparable to our study. A study from Canada determined that several antimicrobial drugs were risk factors for multidrug resistant *Enterobacteriaceae* (*30*). In contrast to our study, most of their isolates had AmpC as a resistance mechanism, and thus their study did not determine risk factors for ESBL-producing bacteria. A 4-year cohort study done in France determined the ESBL-producing bacteria colonization rate in 2 ICUs to be 0.97% and thus concluded that, in their setting, active surveillance was unlikely to be cost-effective (*31*). A study in Israel identified 26 (10.8%) of 241 patients tested by active surveillance as colonized with ESBL-producing bacteria. Risk factors identified in multivariable analysis were poor functional status, current antimicrobial drug use, chronic renal insufficiency, liver disease, and the use of histamine_2_ receptor antagonists (*32*).

A limitation of our study is that we did not have access to records of the antimicrobial drugs that patients may have received as outpatients before their hospital admission. However, relevant to the question of empiric therapy, most intensive care clinicians do not have access to records of outpatient antimicrobial drug use when they are empirically choosing antimicrobial agents. Another limitation of the study is that we did not have access to the subsequent ESBL-positive clinical isolates and thus were unable to compare them by molecular epidemiologic methods, such as pulsed-field gel electrophoresis, to see whether they were identical to the ESBL-colonizing isolates identified previously. We did not perform chart review; thus, the subsequent clinical cultures with ESBL-producing bacteria could have represented either clinical infection or colonization, based on definitions from the Centers for Disease Control and Prevention (*33*). The use of ceftazidime in the screening agar may have caused the CTX-M family of β-lactamases to be missed. However, no CTX-M enzymes were detected in a sample of clinical isolates from the University of Maryland Medical School and the adjacent Veterans Affairs Medical Center from 2001 to 2002 (*34*).

In this study, we identified risk factors for ESBL-producing bacterial colonization among ICU patients. These data may be useful for identifying which patients may warrant empiric ESBL-targeted antimicrobial drug therapy. We also demonstrate that subsequent infections with ESBL-producing bacteria develop in a large percentage of ICU patients colonized with ESBL-producing bacteria.

## References

[1] Lautenbach E, Patel JB, Bilker WB, Edelstein PH, Fishman NO. Extended-spectrum beta-lactamase-producing *Escherichia coli* and *Klebsiella pneumoniae*: risk factors for infection and impact of resistance on outcomes. Clin Infect Dis. 2001;32:1162–71. 10.1086/319757 11283805

[2] Paterson DL. Resistance in gram-negative bacteria: *Enterobacteriaceae.* Am J Med. 2006;119:S20–8. 10.1016/j.amjmed.2006.03.013 16735147

[3] Paterson DL, Ko WC, Von Gottberg A, Mohapatra S, Casellas JM, Goossens H, Antibiotic therapy for *Klebsiella pneumoniae* bacteremia: implications of production of extended-spectrum beta-lactamases. Clin Infect Dis. 2004;39:31–7. 10.1086/420816 15206050

[4] Rossi F, Baquero F, Hsueh PR, Paterson DL, Bochicchio GV, Snyder TA, In vitro susceptibilities of aerobic and facultatively anaerobic Gram-negative bacilli isolated from patients with intra-abdominal infections worldwide: 2004 results from SMART (Study for Monitoring Antimicrobial Resistance Trends). J Antimicrob Chemother. 2006;58:205–10. 10.1093/jac/dkl199 16717055

[5] National Nosocomial Infections Surveillance (NNIS) System Report. Data summary from January 1992 through June 2004, issued October 2004. Am J Infect Control. 2004;32:470–85. 10.1016/j.ajic.2004.10.001 15573054

[6] Blot S, Depuydt P, Vogelaers D, Decruyenaere J, De Waele J, Hoste E, Colonization status and appropriate antibiotic therapy for nosocomial bacteremia caused by antibiotic-resistant gram-negative bacteria in an intensive care unit. Infect Control Hosp Epidemiol. 2005;26:575–9. 10.1086/502575 16018434

[7] Furuno JP, Perencevich EN, Johnson JA, Wright MO, McGregor JC, Morris JG Jr, Methicillin-resistant *Staphylococcus aureus* and vancomycin-resistant enterococci co-colonization. Emerg Infect Dis. 2005;11:1539–44. 16318693 10.3201/eid1110.050508 PMC3366750

[8] Harris AD, Nemoy L, Johnson JA, Martin-Carnahan A, Smith DL, Standiford H, Co-carriage rates of vancomycin-resistant *Enterococcus* and extended-spectrum beta-lactamase–producing bacteria among a cohort of intensive care unit patients: implications for an active surveillance program. Infect Control Hosp Epidemiol. 2004;25:105–8. 10.1086/502358 14994933

[9] Pena C, Pujol M, Ricart A, Ardanuy C, Ayats J, Linares J, Risk factors for faecal carriage of *Klebsiella pneumoniae* producing extended spectrum beta-lactamase (ESBL-KP) in the intensive care unit. J Hosp Infect. 1997;35:9–16. 10.1016/S0195-6701(97)90163-8 9032631

[10] Clinical Laboratory Standards Institute. Performance standards for antimicrobial susceptibility testing, 16th informational supplement (M100–S16). Wayne (PA): the Institute; 2006.

[11] Von Korff M, Wagner EH, Saunders K. A chronic disease score from automated pharmacy data. J Clin Epidemiol. 1992;45:197–203. 10.1016/0895-4356(92)90016-G 1573438

[12] Deyo RA, Cherkin DC, Ciol MA. Adapting a clinical comorbidity index for use with ICD-9-CM administrative databases. J Clin Epidemiol. 1992;45:613–9. 10.1016/0895-4356(92)90133-8 1607900

[13] McGregor JC, Perencevich EN, Furuno JP, Langenberg P, Flannery K, Zhu J, Comorbidity risk-adjustment measures were developed and validated for studies of antibiotic-resistant infections. J Clin Epidemiol. 2006;59:1266–73. 10.1016/j.jclinepi.2006.01.016 17098569

[14] Lipworth AD, Hyle EP, Fishman NO, Nachamkin I, Bilker WB, Marr AM, Limiting the emergence of extended-spectrum Beta-lactamase-producing enterobacteriaceae: influence of patient population characteristics on the response to antimicrobial formulary interventions. Infect Control Hosp Epidemiol. 2006;27:279–86. 10.1086/503016 16532416

[15] Rice LB, Eckstein EC, DeVente J, Shlaes DM. Ceftazidime-resistant Klebsiella pneumoniae isolates recovered at the Cleveland Department of Veterans Affairs Medical Center. Clin Infect Dis. 1996;23:118–24. 8816140 10.1093/clinids/23.1.118

[16] Bartlett JG, Breiman RF, Mandell LA, File TM Jr. Community-acquired pneumonia in adults: guidelines for management. The Infectious Diseases Society of America. Clin Infect Dis. 1998;26:811–38. 10.1086/513953 9564457

[17] Guidelines for the management of adults with hospital-acquired, ventilator-associated, and healthcare-associated pneumonia. Am J Respir Crit Care Med. 2005;171:388–416. 10.1164/rccm.200405-644ST 15699079

[18] Hughes WT, Armstrong D, Bodey GP, Bow EJ, Brown AE, Calandra T, 2002 guidelines for the use of antimicrobial agents in neutropenic patients with cancer. Clin Infect Dis. 2002;34:730–51. 10.1086/339215 11850858

[19] Carratala J, Fernandez-Sabe N, Ortega L, Castellsague X, Roson B, Dorca J, Outpatient care compared with hospitalization for community-acquired pneumonia: a randomized trial in low-risk patients. Ann Intern Med. 2005;142:165–72. 15684204 10.7326/0003-4819-142-3-200502010-00006

[20] Marrie TJ, Lau CY, Wheeler SL, Wong CJ, Vandervoort MK, Feagan BG. A controlled trial of a critical pathway for treatment of community-acquired pneumonia. CAPITAL Study Investigators. Community-Acquired Pneumonia Intervention Trial Assessing Levofloxacin. JAMA. 2000;283:749–55. 10.1001/jama.283.6.749 10683053

[21] Safdar N, Maki DG. The commonality of risk factors for nosocomial colonization and infection with antimicrobial-resistant *Staphylococcus aureus*, enterococcus, gram-negative bacilli, *Clostridium difficile*, and *Candida.* Ann Intern Med. 2002;136:834–44. 12044132 10.7326/0003-4819-136-11-200206040-00013

[22] Thomson KS, Moland ES. Cefepime, piperacillin-tazobactam, and the inoculum effect in tests with extended-spectrum beta-lactamase–producing *Enterobacteriaceae.* Antimicrob Agents Chemother. 2001;45:3548–54. 10.1128/AAC.45.12.3548-3554.2001 11709338 PMC90867

[23] Saurina G, Quale JM, Manikal VM, Oydna E, Landman D. Antimicrobial resistance in *Enterobacteriaceae* in Brooklyn, NY: epidemiology and relation to antibiotic usage patterns. J Antimicrob Chemother. 2000;45:895–8. 10.1093/jac/45.6.895 10837447

[24] Donskey CJ, Chowdhry TK, Hecker MT, Hoyen CK, Hanrahan JA, Hujer AM, Effect of antibiotic therapy on the density of vancomycin-resistant enterococci in the stool of colonized patients. N Engl J Med. 2000;343:1925–32. 10.1056/NEJM200012283432604 11136263 PMC4370337

[25] Donskey CJ, Hanrahan JA, Hutton RA, Rice LB. Effect of parenteral antibiotic administration on persistence of vancomycin-resistant *Enterococcus faecium* in the mouse gastrointestinal tract. J Infect Dis. 1999;180:384–90. 10.1086/314874 10395853

[26] Montecalvo MA, de Lencastre H, Carraher M, Gedris C, Chung M, VanHorn K, Natural history of colonization with vancomycin-resistant *Enterococcus faecium.* Infect Control Hosp Epidemiol. 1995;16:680–5. 10.1086/647041 8683085

[27] Ostrowsky BE, Venkataraman L, D'Agata EM, Gold HS, DeGirolami PC, Samore MH. Vancomycin-resistant enterococci in intensive care units: high frequency of stool carriage during a non-outbreak period. Arch Intern Med. 1999;159:1467–72. 10.1001/archinte.159.13.1467 10399898

[28] Chang FY, Singh N, Gayowski T, Wagener MM, Marino IR. *Staphylococcus aureus* nasal colonization in patients with cirrhosis: prospective assessment of association with infection. Infect Control Hosp Epidemiol. 1998;19:328–32. 10.1086/647823 9613693

[29] Coello R, Glynn JR, Gaspar C, Picazo JJ, Fereres J. Risk factors for developing clinical infection with methicillin-resistant *Staphylococcus aureus* (MRSA) amongst hospital patients initially only colonized with MRSA. J Hosp Infect. 1997;37:39–46. 10.1016/S0195-6701(97)90071-2 9321727

[30] Gardam MA, Burrows LL, Kus JV, Brunton J, Low DE, Conly JM, Is surveillance for multidrug-resistant *Enterobacteriaceae* an effective infection control strategy in the absence of an outbreak? J Infect Dis. 2002;186:1754–60. 10.1086/345921 12447761

[31] Thouverez M, Talon D, Bertrand X. Control of *Enterobacteriaceae* producing extended-spectrum beta-lactamase in intensive care units: rectal screening may not be needed in non-epidemic situations. Infect Control Hosp Epidemiol. 2004;25:838–41. 10.1086/502305 15518025

[32] Ben-Ami R, Schwaber MJ, Navon-Venezia S, Schwartz D, Giladi M, Chmelnitsky I, Influx of extended-spectrum beta-lactamase-producing *Enterobacteriaceae* into the hospital. Clin Infect Dis. 2006;42:925–34. 10.1086/500936 16511754

[33] Garner JS, Jarvis WR, Emori TG, Horan TC, Hughes JM. CDC definitions for nosocomial infections, 1988. Am J Infect Control. 1988;16:128–40. 10.1016/0196-6553(88)90053-3 2841893

[34] Moland ES, Hanson ND, Black JA, Hossain A, Song W, Thomson KS. Prevalence of newer beta-lactamases in gram-negative clinical isolates collected in the United States from 2001 to 2002. J Clin Microbiol. 2006;44:3318–24. 10.1128/JCM.00756-06 16954267 PMC1594717

